# IntuitivePlan inverse planning performance evaluation for Gamma Knife radiosurgery of AVMs

**DOI:** 10.1002/acm2.12973

**Published:** 2020-08-04

**Authors:** Ian Paddick, Diana Grishchuk, Alexis Dimitriadis

**Affiliations:** ^1^ Queen Square Radiosurgery Centre National Hospital for Neurology and Neurosurgery London UK

**Keywords:** Gamma Knife, inverse planning, radiosurgery, treatment planning

## Abstract

**Purpose:**

To compare planning indices achieved using manual and inverse planning approaches for Gamma Knife radiosurgery of arterio‐venous malformations (AVMs).

**Methods and materials:**

For a series of consecutive AVM patients, treatment plans were manually created by expert planners using Leksell GammaPlan (LGP). Patients were re‐planned using a new commercially released inverse planning system, IntuitivePlan. Plan quality metrics were calculated for both groups of plans and compared.

**Results:**

Overall, IntuitivePlan created treatment plans of similar quality to expert planners. For some plan quality metrics statistically significant higher scores were achieved for the inversely generated plans (Coverage 96.8% vs 96.3%, *P* = 0.027; PCI 0.855 vs 0.824, *P* = 0.042), but others did not show statistically significant differences (Selectivity 0.884 vs 0.856, *P* = 0.071; GI 2.85 vs 2.76, *P* = 0.096; Efficiency Index 47.0% vs 48.1%, *P* = 0.242; Normal Brain V_12_(cc) 5.81 vs 5.79, *P* = 0.497). Automatic inverse planning demonstrated significantly shorter planning times over manual planning (3.79 vs 11.58 min, *P* < 10^−6^) and greater numbers of isocentres (40.4 vs 10.8, *P* < 10^−6^), with an associated cost of longer treatment times (57.97 vs 49.52 min, *P* = 0.009). When planning and treatment time were combined, there was no significant difference in the overall time between the two methods (61.76 vs 61.10, *P* = 0.433).

**Conclusions:**

IntuitivePlan can offer savings on the labor of treatment planning. In many cases, it achieves higher quality indices than those achieved by an “expert planner”.

## 1 INTRODUCTION

Treatment planning using multiple isocentres is extremely complex and requires significant experience to become an expert user. Treatment plan quality varies depending on target complexity but also between users.^1^

The treatment planning of arterio‐venous malformations (AVMs) is particularly challenging, as the target nidus is often a highly irregular shape, creating difficulty in achieving conformal plans. The planning process can take a significant amount of clinical time, with the manual placement of multiple isocentres and sector beam blocking used to aid conformity. The method of isocentre placement to create a conformal plan is ideally suited to an automated task which should be able to save significant time while improving the overall quality of treatment.

Inverse planning has been routinely used in conventional radiation therapy for over two decades and has been refined to the point that it is considered mandatory for many treatment planning tasks. While inverse planning has been available in Leksell GammaPlan (LGP) for a similar length of time, it's use has been limited as it has not been able to consistently outperform manual planners that have significant experience. In addition, application of “finishing touches” to a plan is something less suited to inverse planning and typically requires manual editing by an experienced user.^3^

### 1.A. Inverse planning description

IntuitivePlan (Intuitive Therapeutics SA, St‐Sulpice, Switzerland) is a novel inverse planning solution compatible with the Gamma Knife. The treatment planning algorithm is based on the description of the complex Gamma Knife planning task as a convex problem. A precomputation of all possible shots inside the target volume, considering all their possible locations, sizes, and shapes, is followed by solving the convex problem to determine the final treatment plan, that is, calculating which of the pre‐computed shots and with which weights will actually be used.

The optimization settings consist of the prescription dose for target(s), (soft) maximum dose objectives for OAR(s) in the proximity of the target(s), and the optimization types guiding the planning process (“Maximize coverage” and “Maximize selectivity” with optional control of the minimum prescription isodose). The globally convex framework guarantees that the optimal solution (in the mathematical sense) for the given problem is found while beam‐on time is minimized as much as possible.

IntuitivePlan offers tools for final plan “tuning.” Once a treatment plan is calculated, optional interactive direct three‐dimensional (3D) manipulation of the isodose surface with the mouse allows plan adjustment in order to optimize the dose distribution inside or outside the target and to further spare OARs if required.

IntuitivePlan is a stand‐alone third‐party solution, available on a separate workstation, used in conjunction with GammaPlan. Possible clinical workflow was described by Levivier et al.^2^

The purpose of this study was to test the feasibility of IntuitivePlan and compare it with manual approaches for Gamma Knife treatment planning of AVM, using real clinical cases.

## 2 MATERIALS AND METHODS

### 2.A. Patients and treatment

Twenty patients harboring single AVMs were manually planned by expert users (first and last authors, both medical physicists) using LGP. Users were described as “expert,” as they had each completed over 1000 treatment plans. Patients were then re‐planned using IntuitivePlan. The group were a clinically representative patient cohort, each having a single target volume, with 2/20 (10%) of target volumes having proximity to an OAR (brainstem) (Table 1).

**Table 1:** Patient demographics.

Parameter	Mean	Range
Patient age (Years)	37.4	11–68
Gender m/f	8/12	
Target volume (cc)	2.567	0.3–7.5
Prescription dose (Gy)	22.2	18–25
Proportion of targets in proximity to OAR	10%	

Contouring was performed using LGP v11.0 (Elekta Instruments AB), firstly by creating AP and lateral contours on stereotactic digitally subtracted angiography (DSA) images, then delineating the 3D shape of the nidus using T1 and T2 weighted MR images.

### 2.B. Manual treatment planning

The technique of manual planning has been well‐described previously^4^ and requires the placing of multiple isocentre “shots” of different diameters into the target, in order to create a prescription isodose that conforms to the shape of the lesion. For the Gamma Knife Perfexion/Icon, each isocentre has 65535 (4^8^ − 1) sector configuration combinations, which can even make the decision of initial isocentre selection difficult. The treatment plan is further refined by adjusting the isocenter positions in 3D space, the relative weight (dose contribution) of each isocentre and the use of beam blocking, which can also be used to enhance directional gradient to spare OARs.

### 2.C. Inverse planning

During this study, patient data were exported from LGP using DICOM export of MR scans, target, OAR, and skull contours. After import into IntuitivePlan, dosimetric constraints were set up according to the departmental clinical protocol to deliver the prescribed dose to the target, while protecting OARs. The computation options were fixed for all patients to maximize selectivity. A single computation iteration was used for all cases to create clinically acceptable plans. The option to use additional plan manipulation was available, but not deemed necessary by the treatment planners. The resulting shot configuration was imported to LGP for the final dosimetrical calculation, normalization, and evaluation.

### 2.D. Evaluation

In order to compare planning results from both workflows, the following plan parameters were evaluated: treatment planning time, beam on time, number of isocentres, prescription isodose, coverage, selectivity, V_12_, and mean brain dose. In addition, the following indices were used:

The Paddick Conformity Index (PCI)^5^ is defined as:

$$PCI=\frac{TV_{\mathrm{PIV}}^{2}}{TV\times PIV}$$

where TV_PIV_ is the volume of the target covered by the prescription isodose, TV is the target volume and PIV is the prescription isodose volume.

The Gradient Index (GI),^6^ is defined as:

$$GI=\frac{PIV_{50\%}}{PIV_{100\%}}$$

where PIV_50%_ is the volume of 50% of the prescription isodose and PIV_100%_ is the volume of the prescription isodose.

In addition, a relatively new plan quality metric, the Efficiency Index (EI), which considers the ratio of integral dose inside vs outside the target, was considered in the evaluation.^7^

$$Efficiency\;Index=\frac{\text{“}Useful\;Energy\text{”}}{\text{“}Total\;Energy\text{”}}=\frac{Integral\;Dose_{\mathrm{TV}}}{Integral\;Dose_{PIV50\%}}=\frac{\int_{D\min}^{D\max}TV\;\delta dose}{\int_{PIV50\%}^{D\max}V\;\delta dose}$$

where D_min_ is the minimum dose to the target, Dmax is the maximum dose to the target, TV is the target volume, and PIV50% is the volume of 50% of the prescription dose. This parameter needs to be manually calculated from dose volume histograms exported from GammaPlan but is automatically calculated by IntuitivePlan. A paired t‐test was used to check for statistical significance between plan parameters of manual and inverse plans.

## 3 RESULTS

**Fig. 1:**
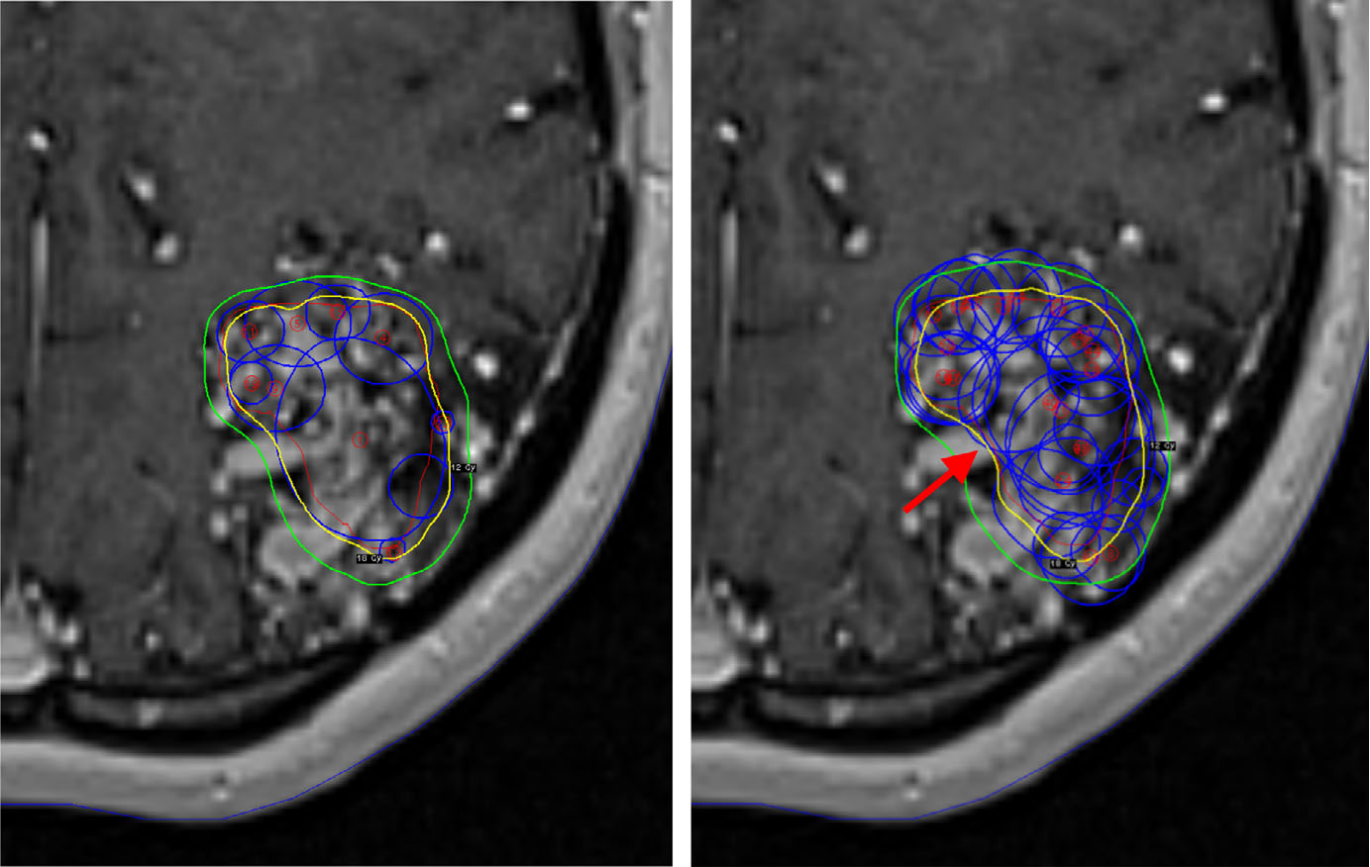
Transaxial T1 weighted magnetic resonance imaging slices with the manual (left) and inverse (right) plans for case number 6. The blue circles denote the isocentre size and position. The 18 Gy (prescription dose) and 12 Gy isodose lines are shown in yellow and green respectively. The improved concavity of the prescription isodose (red arrow) on the medial edge of target is shown on the inverse plan.

Figure 1 shows an example of manual and inverse plans for case number 6 in LGP showing all isocentres. Imported plans created by IntuitivePlan present themselves like all other plans in LGP and theoretically could be modified in that environment if desired. Apart from slight renormalizations (changes in the prescription isodose), plans have been used as calculated by IntuitivePlan.

**Fig. 2:**
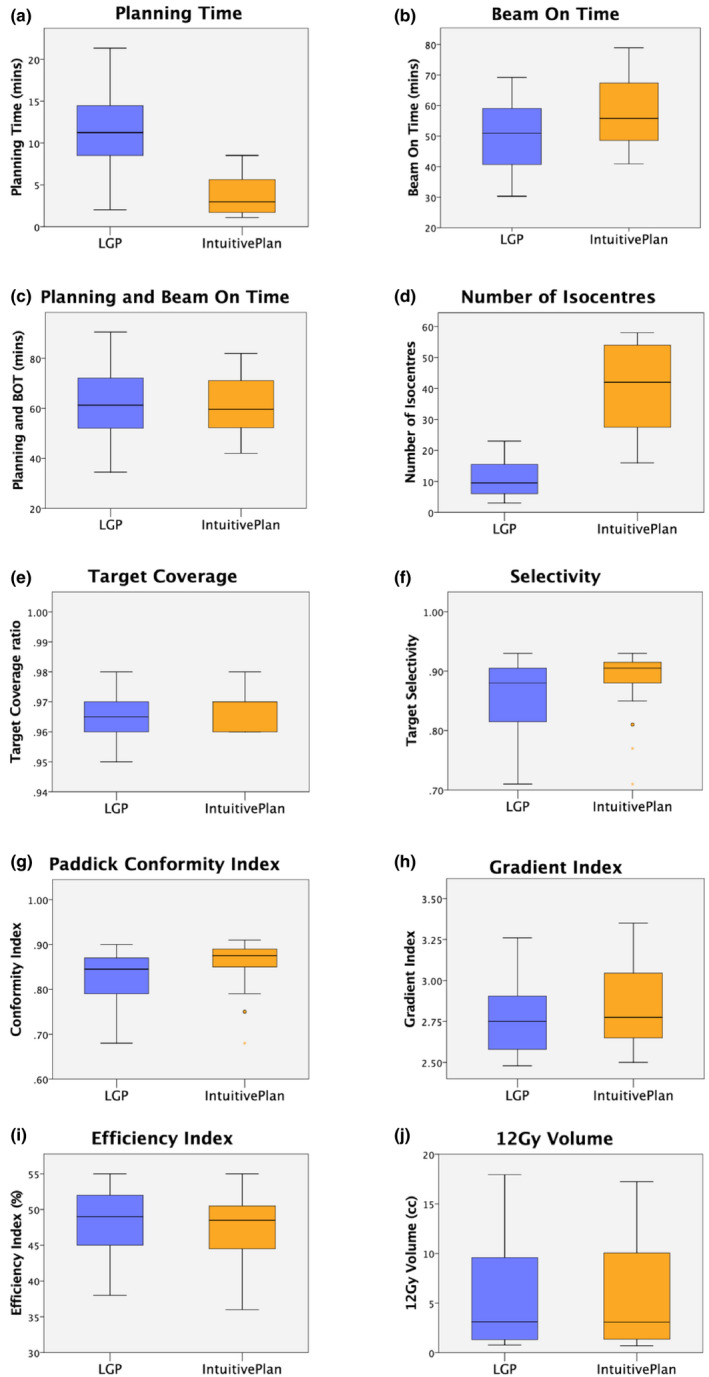
Box plots showing planning parameters for the 20 cases: (a) Planning time; (b) Beam on time; (c) Planning and beam on time; (d) Number of isocentres; (e) Target coverage; (f) Selectivity; (g) Paddick Conformity Index; (h) Gradient Index; (i) Efficiency Index; (j) 12 Gy volume.

Numerical results for manual and inverse plans together with p‐values for the paired t‐tests are summarized in Table 2 and presented graphically on Fig. 2. Considering mean planning parameters, IntuitivePlan demonstrated significantly shorter planning times (3.79 vs 11.58 min, *P* < 10^−6^) but longer beam‐on times (57.97 vs 49.52 mins, *P* = 0.009). When these parameters were combined, there was no significant difference in the total planning and beam‐on time: (61.8 vs 61.1 mins, *P* = 0.433).

**Table 2:** Mean values of manual and IntuitivePlan plan parameters. *P*‐values in bold if <0.05.

Parameters	Manual plan	Intuitive Plan	*P*‐value (paired *t*‐test)
Planning time (min)	11.58	3.79	**>10^‐6^**
Beam on time (min)	49.52	57.97	**0.009**
Planning plus beam on time (min)	61.10	61.76	0.433
Number of isocentres	10.8	40.4	**>10^‐6^**
Prescription isodose (%)	45.35	50.85	**0.017**
Coverage (%)	96.3	96.8	**0.027**
Selectivity	0.856	0.884	0.071
Paddick conformity index	0.824	0.855	**0.042**
Gradient index	2.76	2.85	0.096
Efficiency index (%)	48.1	47.0	0.242
Normal brain V_12_ (cc)	5.79	5.81	0.497
Mean brain (Skull) dose (Gy)	0.35	0.35	0.500

Inverse plans used a larger number of isocentres: (40.4 vs 10.8, *P* < 10^−6^), had higher target coverage (96.8% vs 96.3%, *P* = 0.027), and a higher PCI (0.855 vs 0.824, *P* = 0.042). Other parameters that did not have a statistically significant difference included selectivity (0.884 vs 0.856, *P* = 0.071), Gradient Index: (2.85 vs 2.76; *P* = 0.096), Efficiency Index (47.0% vs 48.1%; *P* = 0.242), and 12 Gy volume (5.81 vs 5.79 cc; *P* = 0.497).

As there were only two targets abutting OARs, no statistically significant difference in OAR dose could be demonstrated, however, IntuitivePlan plan showed slightly higher, but clinically acceptable doses to the OAR (Brainstem V_12_ = 0.1 cc).

## 4 DISCUSSION

Historically, treatment planning for Gamma Knife used forward, or manual planning by a team of neurosurgeons, radiation oncologists, and physicists. However, plan quality produced using forward/manual planning depends very much on planner experience, being complex and often counterintuitive. This is due to the nature of the method, which is a very sophisticated problem. Compared with conventional radiotherapy treatment planning the goal is not only to get a clinically acceptable plan, but also an efficient one in terms of treatment delivery ie. limiting overall treatment time.

The “Wizard” feature became available in LGP v 5.34 in 2000 and was the first commercially available inverse planning tool for the Gamma Knife. However, this primitive tool was often considered by expert planners to produce inferior results; placing isocentres too far outside the target, and due to its own limitations, not respecting OARs.^8^ It was not until the concept of the Gradient Index^6^ became accepted that an inverse planning tool was developed that could consider the gradient outside the target (LGP v 10, 2010). This version of the inverse planning software could potentially streamline the clinical workflow, particularly when applied to planning large, rounded targets in non‐eloquent areas.^3^ However, for complex targets, manual planning is usually preferred, particularly by expert planners^9^.

This is the first study evaluating the performance of IntuitivePlan for the planning of AVM. This is the first commercial third‐party planning solution for Gamma Knife, which, since its first clinical version (v.1.0.0),^1^ has had the addition of plan adaptation tools as well as treatment delivery time optimization; enabling a shorter calculation time by its optimization framework, which optimizes isocentre location, collimator configuration and weighting in parallel.

In order to test IntuitivePlan clinically for AVM radiosurgery, we used planners with a long‐term experience of manual planning, having each planned over 1000 cases. The experience/ability of the treatment planner in this sort of study represents a potential bias and results might be different if manual plans are created by less experienced users. All manual plans were optimized until they were clinically acceptable, with quality being maximized within what was considered to be a reasonable time. Further improvement is always possible but this was not pursued in order to balance the trade‐off between planning time and plan quality.

Importantly, planning with IntuitivePlan commenced after brief application training (approx. 1 h) and clinically acceptable plans were achieved in “Maximize selectivity” mode for all cases after the first calculation iteration. The “Maximize coverage” optimization option in Intuitive Plan results in 100% coverage of the target volume at the expense of selectivity. This is difficult to clinically justify for benign disease, such as AVMs, but may be of value in the planning of malignant disease. Changing the modification parameters and 3D manipulation of the isodose surfaces were not used during this study. This would have significantly increased planning time, because the plan has to be recalculated after each manipulation. That is why for the purpose of this study, the option “Maximize selectivity” was used for all patients. This resulted in plans with a selectivity with no significant difference from the manual plans but with a significantly higher coverage and PCI, and longer treatment time. Those indices are not fully independent. We expect that the longer treatment time is due to the higher PCI achieved.

In this study, planning time is shorter for IntuitivePlan. During the calculation there is no need for the user to interact with the system, so this time can be used for other tasks. By contrast, the treatment delivery time was found to be longer for plans generated by inverse planning compared to manual planning. However, when combined, the ‘Planning’ plus ‘Beam On’ time is similar for both systems.

As IntuitivePlan is separate from, but reliant on LGP, for the creation of the patient file as well as for the final evaluation and export of the treatment plan, there is an additional time penalty for transfer of the data between the two systems. This transfer uses a USB memory stick and takes approximately 3 min in total. In this work, data transfer time was not taken into account, but when added to the inverse planning workflow, it did not change the statistical significance of the overall time difference. If, in the future, IntuitivePlan was incorporated within the LGP planning software, transfer time would not be required.

Isocentre configuration and the prescription isodose line are optimized by IntuitivePlan and cannot be manually adjusted before export to LGP. This selection of an optimal isodose can help enhance the quality of the plan, as the optimal prescription isodose has been shown to vary depending on the individual treatment plan.^6^ When the shot configuration is recalculated by LGP, the plan undergoes recalculations with minor renormalization. At this point the user can adjust the prescription isodose, balancing target coverage, and selectivity. This may mean that selecting a particular isodose with a particular level of target coverage (e.g., 99% coverage of the target with the 50% isodose) is not possible without some manual readjustment of the plan. However, planning to a particular isodose has not been shown to be an important factor for clinical effect, and there is little to no evidence that the maximum dose impacts on the efficacy or safety of SRS treatments. ^10^, ^11^

Like any inverse planning system, OARs should be contoured in order to set up dose constraints. This can potentially increase contouring time since for most manual planning scenarios, OARs are not contoured routinely. Moreover, some centres do not contour target volumes, which is an obvious requirement for any inverse planning system. Another important step prior to planning is contour quality control. All contours should be smooth, consistent and not overlap with each other. Imperfections may impact on both manual and inverse planning results. However, IntuitivePlan has a contour smoothing tool which may even out minor contouring inconsistencies. The possibility of direct manipulation of the isodose surface allows the planner to increase and/or relax constraints, even if an OAR has not been contoured.

Figure 1 shows the example of the manual and IntuitivePlan treatment plans for case number 6 in GammaPlan with all isocentres (shots) displayed. The large number of isocentres used in an IntuitivePlan may appear alarming to experienced Gamma Knife users who are often trained to avoid using too many isocentres. This is partly because more isocentres are associated with a longer beam on time. Despite IntuitivePlans having on average almost four times as many isocentres, the beam on time was only 18% higher. Overall treatment time, which includes movement between isocentres has not been evaluated. For the Gamma Knife Perfexion/Icon, movement takes approximately 3 s between each isocentre, so the increase in the mean number of isocentres from 10.8 for the manual plan to 40.4 for the IntuitivePlan corresponds with a modest increase of just under one and a half minutes in treatment delivery time. Often, the use of a large number of small isocentres can lead to a better dose gradient, but in this study we did not see this effect. The GI was slightly worse (larger) for the IntuitivePlan plans, though this was not statistically significant.

An increase in treatment time is known to reduce the biologically effective dose (BED)^12^, and this effect has now been demonstrated in clinical series of Gamma Knife patients. This impact was not evaluated in this study. Whilst both methods may have achieved similar levels of conformity and gradient and delivered the same physical dose, the BED might be significantly different between competing plans. Future inverse planning solutions should consider developing BED‐based optimizations to ensure that regardless of the treatment delivery time, the plans generated have the same BED.^13^, ^14^

Plan quality metrics are better for IntuitivePlan in general, but the difference is small and might not have clinical relevance. The 12 Gy volume, which was the very first radiosurgery plan quality parameter that was demonstrated to have a clinical consequence was nearly equal using both planning methods. Furthermore, the mean skull dose (the structure, automatically constructed by LGP and used as a surrogate for mean brain dose) was identical for the two planning systems.

As there were only two targets abutting OARs, performance in protecting structures could not be properly evaluated. However, both OARs received doses below tolerance. The balance between OAR dose and other planning parameters is complex and achieving an even lower OAR dose may have adversely affected other parameters, confounding our results.

A small difference in coverage was noted between the two planning methods. It could have been possible to renormalize the plans so that near identical coverage was achieved. One could argue that this would allow fairer comparison between the two methods. However, coverage is normally inversely balanced with selectivity and the IntuitivePlans had both higher coverage and selectivity.

## 5 CONCLUSIONS

IntuitivePlan can offer savings on treatment planning times. In many cases, it achieves what the authors consider to be better than “expert planner” quality. These qualities may also yield significant time savings, particularly for inexperienced users.

The use of IntuitivePlan can free the user from the labor of forward planning and may offer more time for actual plan optimization. Because the user does not need to interact with the system during calculation times, this time can be used for other tasks.

A number of innovative advantages including contour smoothing, EI calculation make it a useful planning tool for the Gamma Knife.

## CONFLICTS OF INTEREST

Ian Paddick works as an ad‐hoc consultant for Elekta.
